# Update on vesicovaginal fistula: A systematic review

**DOI:** 10.1080/2090598X.2019.1590033

**Published:** 2019-04-04

**Authors:** Ahmed S. El-Azab, Hassan A. Abolella, Mahmoud Farouk

**Affiliations:** Department of Urology, Assiut University Urology Hospital, Assiut University, Assiut, Egypt

**Keywords:** Fistula, vesicovaginal fistula, genitourinary fistula

## Abstract

**Objective**: To conduct a systematic review of the literature on vesicovaginal fistula (VVF), including reporting on the aetiology, in both developed and underdeveloped countries; diagnosis; intraoperative prevention; and management.

**Methods**: We conducted a systematic review of the literature on VVF through the PubMed and the Cochrane Library according to the Preferred Reporting Items for Systematic Reviews and Meta-Analyses (PRISMA) statement. The search was conducted from 1985 to 2018 in English, using the keywords ‘fistula’ and ‘vesicovaginal fistula’. Prospective studies were preferred; however, retrospective studies and case reports were used when no prospective studies were available. All authors’ extracted relevant data related to the proposed review of VVF and carefully examined collected articles.

**Results**: In all, 116 relevant articles were identified and 43 articles were included in this systematic review. The outcome of surgical reconstruction was >90%, but the outcome may be suboptimal in radiotherapy (RT)-induced VVFs. Absolute indications for an abdominal approach included: ureteric involvement, the need for concomitant bladder augmentation, severe vaginal stenosis, and an inability to tolerate the dorsal lithotomy position (e.g. due to muscular spasticity). Typically, it was recommended to wait at least 3 months to allow the inflammatory response to subside before definitive surgery. Early fistula repair can be performed in the absence of infection and in patients who have not received pelvic RT.

**Conclusion**: VVF is rare in developed countries. Surgical treatment is the primary method of repair. The outcome of surgical reconstruction exceeds 90%, but the outcome may be suboptimal in RT-induced VVFs.

**Abbreviations:** PRISMA: Preferred Reporting Items for Systematic Reviews and Meta-Analyses; RT: radiotherapy; (S)UI: (stress) urinary incontinence; UVF: ureterovaginal fistula; VVF: vesicovaginal fistula

## Introduction

Vesicovaginal fistula (VVF) is an abnormal epithelialised or fibrous connection between the bladder and vagina, which results in continuous and unremitting urinary incontinence (UI). VVFs are rare in developed countries and arise mainly from malignant disease, radiotherapy (RT), or surgical trauma [1]. VVF is a debilitating condition for women not only in developing countries but for women in all parts of the world. Herodotus noted the continuous leakage of urine after difficult labour. Avicenna, a Persian physician, documented the relationship between VVFs and obstructed labour in 1037 AD [2]. At least 3 million women worldwide, most of them in Africa and southern Asia, have an untreated VVF, whilst between 30 000 and 130 000 new VVFs develop annually in Africa alone [3].

Most reviews on VVF reported on obstetric fistula [4] or reviews in certain localities [5,6]. Studies on VVF were not specific, i.e. answering a general question. A review of the existing reports on VVFs also concluded that the literature consists mainly of case series and personal experiences. In the present review, we carried out a systematic review on carefully selected up-to-date articles, including those reporting on the aetiology of VVF, in both developed and underdeveloped countries. In the present review, we discuss the incidence of VVF, review the literature on the aetiologies, predisposing factors and diagnosis; and describe treatments, including preoperative, intraoperative, and postoperative care. Intraoperative prevention and management is also discussed in some detail.

## Methods

**Figure 1.**
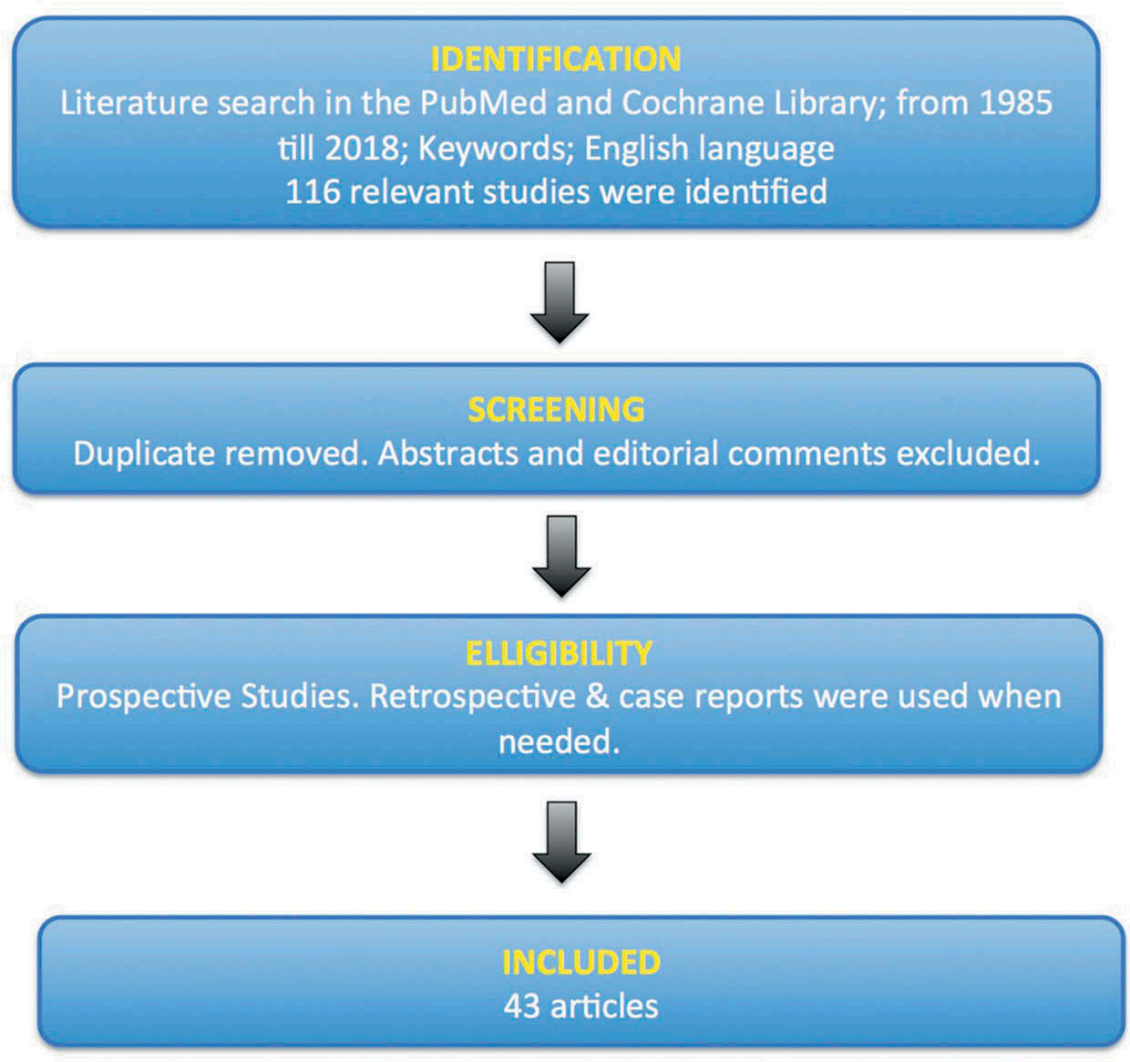
Flow chart for the selection of the studies in our systematic review according to the PRISMA statement.

We conducted a systematic review through the PubMed and Cochrane Library according to the Preferred Reporting Items for Systematic Reviews and Meta-Analyses (PRISMA) statement [7]. The search included the period from 1985 to 2018, using the keywords ‘fistula’ and ‘vesicovaginal fistula’. All authors extracted relevant data related to the proposed review of VVF by carefully examining the collected articles. We limited our search to studies related to the epidemiology, aetiology, intraoperative prevention, and treatment of VVF. Prospective studies were preferred; however, retrospective studies and case reports were used when no prospective studies were available. After applying these criteria, a total of 116 papers were identified. The authors then evaluated these articles based on study design, number of patients, and presence of relevant information in the study. Finally we identified 43 articles according to our search criteria that were included in our systematic review (Figure 1).

## Results

In all, 116 relevant articles evaluating the epidemiology, aetiology, intraoperative prevention, and treatment of VVF were identified. Duplicate studies and abstracts were excluded. Only full-text articles in the English language were included. Few studies were designed to answer a specific clinical question, with most studies answering a general question. As mentioned before, prospective studies were preferred. However, retrospective studies and case reports were also included when no prospective studies were available. Of the 116 articles, 43 articles were included in our qualitative analysis based on our inclusion criteria. All articles were analysed and divided into groups according to the question posed.

### Aetiology

VVF is much less common in developed countries, where it arises mainly as a complication of pelvic surgery (e.g. hysterectomy) or RT for cancer. Hillary et al. [8], in their systematic review, reported that 83.2% of cases of VVF in developed countries had a surgical aetiology (e.g. simple abdominal hysterectomy and other types of pelvic surgery, including benign and malignant colorectal, urological, and gynaecological procedures), whilst only 4.8% were of a surgical aetiology in underdeveloped countries. VVFs following abdominal hysterectomy account for 75% of all fistulae. The precipitating factor is mostly unnoticed injury to the bladder during surgery or inadvertent placement of a suture or a clamp into the bladder wall. It is estimated that 0.5–2% of hysterectomies are complicated by VVFs [9].

Obstetric VVF due to obstetric trauma is common in underdeveloped countries [8]. Of VVFs reported from underdeveloped countries, 95.2% of cases were of an obstetric aetiology, mostly due to prolonged neglected obstructed labour. In 9% of cases VVFs followed caesarean section and 2% following instrumental delivery [8]. VVF results from prolonged obstructed neglected labour with subsequent ischaemic pressure on the anterior vaginal wall and the base of the bladder during prolonged labour [10]. The major risk factor appears to be prolonged obstruction that produces an extended period of ischaemia of the bladder and vaginal wall that leads to tissue necrosis and the subsequent development of a VVF.

RT-induced VVF is a special challenge to the urologist. Failure rates after repair of these fistulae are as high as 50% because tissues are often poorly vascularised [11]. The bladder is commonly fibrotic and non-compliant and sometimes requires augmentation [12]. VVFs that develop after RT may manifest months to years after and are associated with endarteritis obliterans and tissue ischaemia [13].

### Diagnosis

The physician should become suspicious of the presence of a VVF when the patient complains of a leakage of urine after a pelvic operation. Occasionally these postoperative VVFs may not develop until a few weeks or even few months after an operation or RT. On pelvic examination, the vagina should be carefully inspected using a speculum; under anaesthesia, if required. Ghoniem and Warda [14] in their review stated that acute VVFs are usually not palpated but by inspection with the speculum, the mucosa surrounding the VVF may appear erythematous and inflamed. However, in mature VVFs an opening is usually seen or palpated in the vagina. A phenazopyridine test can be performed by giving the patient oral phenazopyridine (pyridium). A vaginal pack or a tampon is inserted into the vagina before taking the phenazopyridine. After careful removal, if the pack reveals the presence of orange stain, there is a high likelihood that a VVF exists. The authors have been using a methylene blue test for many years, with very good sensitivity. The test is carried out by installing 100 mL methylene blue solution into the bladder through the urethra using a catheter. After removing the catheter, three cotton swabs are placed into the vagina. After 2 h the swabs are inspected and if stained blue this indicates a VVF; whilst an orange stain indicates a ureterovaginal fistula (UVF) [14].

### Radiological examinations

**Figure 2.**
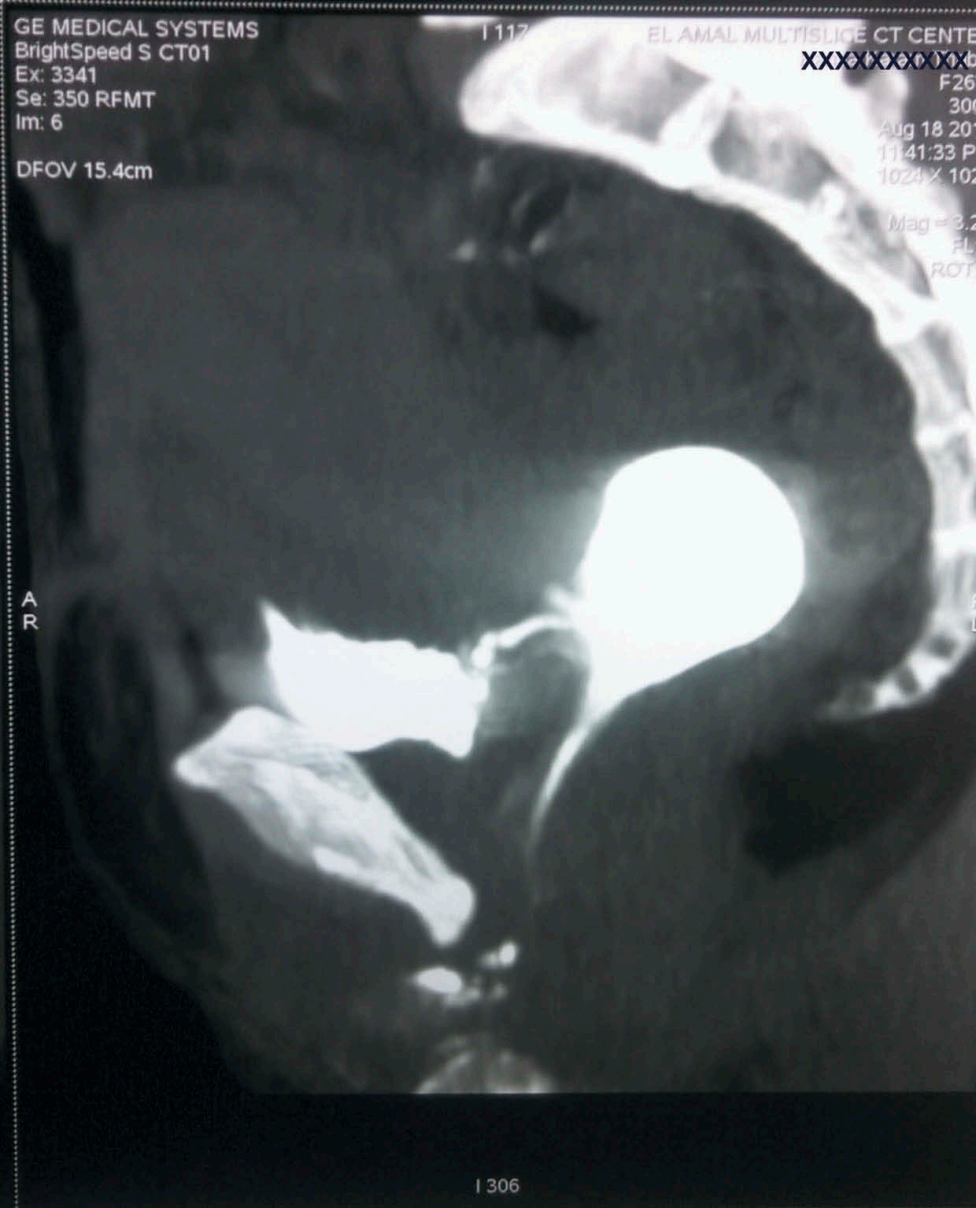
Lateral CT cystogram with contrast demonstrating VVF.

IVU is useful for excluding UVFs, which are present 10% of the time with VVFs. Findings at IVU that may suggest ureteric involvement include: hydronephrosis, extravasation of the dye, or a persistent column of contrast in the ureter [12]. CT findings suggestive of VVF include: a CT finding of contrast within the vagina, detection of air and/or fluid within the vagina, lateral cystograms may show the tract (Figure 2). CT may also identify the cause of the fistula such as: RT changes, contiguous pelvic mass, or adherent thickened bowel. CT also provides important information regarding the surgical field and the extent of disease before attempted surgical repair [15].

### Cystoscopy

Cystoscopic examination confirms the presence of the VVF, determines the presence of additional fistulae, and assesses its size and location in relation to the ureteric orifices. When the opening of the fistula is unidentifiable, passage of a guidewire through the fistula may help identification. Biopsy from the site of the VVF should be taken at the time of cystoscopy in cases of malignant or RT-induced fistulae. Ascending ureteropyelogram can be performed if the surgeon still suspects ureteric involvement [16]. Finally, the methylene blue test can be performed again during cystoscopy.

### Intraoperative prevention

Bladder injury during vaginal surgery creates the potential for the development of a postoperative VVF. To prevent the formation of fistulae, further dissection is required. Adequate mobilisation and exposure not only will provide for a tension-free closure, it will also help determine if there are any other bladder injuries. Proper anatomical closure of the bladder opening should be performed in two layers. Proper postoperative bladder drainage is also essential preferably by both a wide-bore urethral catheter and suprapubic catheter. The original operation can be continued in the absence of any infections and provided that the procedure does not incorporate a synthetic material adjacent to the cystotomy closure such as transobturator tape or artificial urinary sphincter [17]. Before catheter removal a cystogram at 7–21 days postoperatively should demonstrate no contrast extravasation.

When the urologist is requested to work with the gynaecologist in pelvic vaginal surgery, e.g. for an anti-incontinence procedure following a vaginal hysterectomy or other vaginal operations such as prolapse, the urologist should perform an initial cystoscopy to assure an intact bladder. If cystoscopy reveals a bladder injury, appropriate repair should be performed prior to the anti-incontinence procedure. Whether or not to continue to implant a synthetic sling is a matter of debate [18].

Occasionally, the urologist is requested for an intraoperative consultation for an unplanned cystotomy during an abdominal operation, e.g. total abdominal hysterectomy. The bladder should be closed in two layers. The suture line should not be placed over other suture lines (i.e. vagina, colon, and others). Interposition of viable tissue should be considered.

Regardless of the operative approach (vaginal or abdominal), the urologist performing an intraoperative consultation for an unplanned cystotomy must ensure that the ureter has not been inadvertently injured. Establishing that the ureters have not been injured or incorporated into any sutures, intraoperative passing of a ureteric catheter should be performed. If the ureters are intact but concerns persist about their postoperative status, the placement of double-pigtail ureteric catheters for 2–12 weeks may be prudent [18].

### Management

The typical current practice is to perform a delayed repair following a period of catheter drainage to allow necrotic tissue to slough and local inflammatory responses to subside [19,20]. The literature describes conservative management for small VVFs uncomplicated by ischaemia, RT, or malignancy. Continuous urethral catheter drainage plus oral antimuscarinics and antibiotics have been associated with a 11% and 15% closure rate, respectively [17]. The exact closure rates from this conservative management are likely to be underestimated given that successful outcomes in this context are frequently unreported [8]. Hilton [21] reported, in a small series of 24 patients, spontaneous successful closure in 6.9% of patients following 6–8 weeks of catheter drainage. Spontaneous closure was never encountered in any case of RT-induced VVFs. In other series, successful conservative management was achieved in 15% of patients [22].

A trial of de-epithelialisation of the fistulous tract has been tried using silver nitrate, mechanical curettage, electrocautery, or laser therapy [23]. The use of a synthetic substance for VVF closure has been tried such as the fibrin glue, bovine collagen and cyanoacrylate glue [24]. Most of these therapies have proven to be ineffective and most cases of VVF will ultimately require surgery for definitive cure [25].

### Surgery

Surgical treatment is the primary method for repairing VVFs. Whether the approach is vaginal or abdominal, the outcome of surgical reconstruction is good and exceeds 90%. The surgeon must be aware that the outcome may be suboptimal in certain types of VVFs, e.g. RT-induced, longstanding (bladder is defunctionalised for a long time), and recurrent. Absolute indications for an abdominal approach include: ureteric involvement, the need for concomitant bladder augmentation, severe vaginal stenosis, and an inability to tolerate the dorsal lithotomy position (e.g. due to muscular spasticity). When the VVF is close to the bladder neck, preoperative documentation of stress UI (SUI) is required. Synthetic slings should be avoided and autologous slings can be used.

Typically it is recommended to wait at least 3 months to allow the inflammatory response to subside before definitive surgery. Early VVF repair can be performed in the absence of infection and in patients who have not received pelvic RT. Contraindications to early repair include: RT-induced VVF and associated enteric injury [26]. The advantage of early repair includes avoidance of prolonged urine leakage, which has a negative effect on the patient’s quality of life. During the waiting period, risk factors for poor healing (malnutrition, RT, immunosuppression, or vaginal atrophy) should be assessed and corrected when possible [27].

### Vaginal approach

Most VVFs are accessible via a transvaginal approach. The vaginal approach is associated with less morbidity, less blood loss, less burdensome for patients, and lesser hospital stay than the abdominal approach. Through the anterior vaginal wall, the vagina is dissected off of the bladder followed by a multilayer closure. Before we start the procedure we insert a ureteric catheter. To ensure bladder drainage, we place both a urethral and suprapubic catheter. We usually use only a wide-bore urethral catheter in cases of straightforward obstetric VVFs [28]. An alternative approach is the Latzko technique. The Latzko technique may be typically indicated for proximal post-hysterectomy VVF. The technique consists of a circumferential ellipsoid incision around the VVF, with wide mobilisation of the vaginal epithelium in all directions. The vaginal epithelium around the VVF site is excised and the fistulous tract is closed. The repair is reinforced by a layer derived from the perivesical tissue. A modified colpocleisis is performed, with several layers of absorbable sutures from the anterior to posterior vaginal wall obliterating the upper vagina. The Latzko partial colpocleisis procedure is an alternative technique to traditional vaginal repair. Shortening of the vaginal canal can occur but rarely affects sexual function. However, caution should be exercised when considering it in sexually active females [29].

### Abdominal approach

Traditionally, the abdominal approach has been indicated in patients who have VVF, or those who require additional intra-abdominal procedures, or simultaneous urological procedures, such as ureteric re-implantation or augmentation cystoplasty. The suprapubic approach described by O’Conor et al. [30] involves bivalving the bladder from the dome to fistulous opening separating the bladder from the vagina for a distance of 2–3 cm beyond the VVF, which is the key step for successful repair. In the transvesical approach, the bladder is opened but not bivalved and the VVF is accessed from inside the bladder, allowing excision of the VVF, dissection between the bladder and vagina, and closure of vagina and bladder. A posterior wall bladder flap may be used to close a large gap or to avoid overlapping of suture lines. A combined transabdominal and transvaginal approach may be used for large, complex or recurrent cases.

### Laparoscopic approach

Nezhat et al. [31], in 1994, were the first to describe a laparoscopic approach to VVF repair, whilst Melamud et al. [32] reported on the first robot-assisted repair of a VVF in 2005. The laparoscopic approach has the advantages that the pneumoperitoneum facilitates dissection of tissue planes, the magnification offered by the video camera can improve visualisation of the tissue, and that patient morbidity and hospital stay are decreased as compared to open surgery [32].

### Graft interposition

Graft interposition is not indicated in all cases of VVF repair [33]. No high-quality evidence supports the routine use of graft interposition. The use of grafting in obstetric VVF has significantly declined [34]. Relying on watertight, tension-free, uninfected multilayer closure is often sufficient. Graft interposition is indicated in cases of recurrent, RT-induced, and long-standing VVFs [35]. A variety of grafts have been used in abdominal repairs including omentum and peritoneum covering the bladder dome [36]. When operating transvaginally, the peritoneal reflection of the cul-de-sac or the more popular Martius bulbocavernosus muscle/fat graft may be interposed between the bladder and vagina to help prevent re-fistulisation [37]. The Martius flap is derived from the labial fat pad and can be based on either the anterior or posterior circulation (pudendal or epigastric) depending upon the location of the lesion to be covered. The flap can be tunnelled under the labia minora to the site of fistula reconstruction. A 0.64 cm (0.25 inch) Penrose drain at the end of the procedure is essential to avoid haematoma collection. Women should be counselled that the donor labial site will appear to be somewhat deformed after harvest but that within 6 months new adipose tissue will correct any cosmetic abnormality. Chromic suture is best for skin closure to avoid prolonged vaginal discharge. The labial fat-pad graft can be used in all areas of the vagina; however, very proximal apical lesions may be difficult to reach with this particular graft and in this case the peritoneal flap would be useful. The peritoneal flap was first described by Raz et al. [11] and involves dissecting the posterior vaginal wall flap posteriorly toward the cul-de-sac. The pre-peritoneal fat and peritoneum are sharply mobilised caudally. The peritoneal flap can then be advanced over the repair and secured with interrupted 3–0 polyglactin 910 (Vicryl®; Ethicon Inc., Somerville, NJ, USA). One study mentioned a higher success rate in a small series of RT-induced VVFs when interposition grafts were used (100%) vs 67% when no grafts were used, although these differences were not statistically significantly different [38].

### Outcome after surgery

Of patients undergoing surgical closure, the median (range) overall closure rate in the literature in industrialised countries is 94.5 (75.8–98.6)%. The median (range) SUI rate after successful VVF repair is 6.5 (1.1–51.9)%, mostly following transvaginal repair of UVFs [8,39]. Amongst patients undergoing surgical repair in underdeveloped countries, the median (range) overall closure rate was 87 (58.0–100)%, whilst the median (range) SUI rate was 10.0 (3.8–30.0)% [8].

There are no randomised studies that compare the outcomes of transabdominal vs transvaginal approaches. Nonrandomised studies compared the outcomes of transvaginal vs transabdominal approaches for fistula repair [21,40,41]. The overall success rate for a vaginal approach was 91% vs 84% for abdominal repairs (*P* = 0.018).

## Discussion

A review of the literature on the issue of VVFs demonstrated that most studies were old and relatively uncritical by current scientific criteria. This literature consists mainly of case series and personal experiences reported by urologists or gynaecologists. The exact magnitude of the problem of VVF in developing countries is, therefore, still unknown. Review of the available evidence suggests that this problem is both enormous and neglected in these countries.

Our present review showed that VVF is extremely rare nowadays in developed countries, where it results mainly from surgical intervention. VVFs are still common in developing countries and in 95% of cases result from obstetric causes. Obstetric VVFs result from prolonged neglected obstructed labour, where sustained pressure leads to ischaemia and necrosis due to compression of the bladder base and anterior vaginal wall between the foetal head and symphysis pubis [3]. Iatrogenic injury may occur during caesarean section or any pelvic surgery. We found in the present review that 76% of VVFs in developed countries resulted from simple hysterectomy. VVFs after pelvic surgery result from inadvertent bladder injury or tissue devitalisation due to extensive dissection or haematoma formation. VVFs that result from pelvic RT are a special challenge to the urologist and may present many months to years later and are associated with extensive ischaemia. Failure rates after repair of these RT-induced VVFs are as high as 50% because tissues are often poorly vascularised. Tissue interposition is mandatory in RT-induced fistulas [11].

Spontaneous closure of the VVF should be attempted. The rate with which this occurs is likely to be underestimated [42]. Management consists of a 6–8-week period of continuous catheter drainage, antibiotics and anticholinergics to allow urine diversion and spontaneous closure before epithelialisation of the fistula track. The reported rate of spontaneous closure ranges from 11% to 15% [17]. RT-induced VVFs, however, are seldom if ever associated with spontaneous closure.

Immediate vs delayed repair: is an issue of debate. The exact definition of ‘immediate’ repair varies between authors, with most considering early repair as at <6 weeks of creation. Waaldijk [22] using a definition of ‘immediate’ as within 3 months of creation, reported a 95.2% successful initial closure rate. Typically, repair should be performed following a period of catheterisation to provide the opportunity for spontaneous closure. However, immediate repair alleviates the patient’s distress. We do not have any strong evidence to support the advantage of immediate repair over a delayed repair; however, it is certainly a challenge to perform a repair between the third week and the third month following VVF formation.

Vascularised tissue flaps or grafts are used to reinforce a repair, fill dead space, and to improve vasculogenesis following a repair. Graft interposition is not indicated in all cases of VVF repair. No high-quality evidence supports the routine use of graft interposition. However, they are definitely indicated in complex, RT-induced, recurrent, and long-standing VVFs. Successful repair of VVF depends on the integration of several preoperative, intraoperative, and postoperative factors. In the authors’ experience, these factors are listed in Table 1. Postoperative care of these cases is essential for optimal outcome. At our centre, the routine postoperative care after surgery for VVF consists of:

1. The vagina is packed with an oestrogen-impregnated vaginal pack for several hours postoperatively for haemostasis.
2. Ensure the bladder is well drained even when patient is discharged home.
3. Prescribe antimuscarinics to avoid postoperative bladder spasm that may interfere with healing. If the urethral catheter is obstructed or if the patient experiences bladder spasms refractory to antimuscarinics, the urethral Foley may be removed, leaving the suprapubic tube only for drainage.
4. We use antibiotics as surgical prophylaxis and in the immediate 24 h after surgery. Prolonged use of antibiotics in the early postoperative period is of no value. However, we give a single dose of antibiotic at the time of removal of catheters to sterilise urine [43].
5. Topical oestrogen promotes healing especially in postmenopausal women.
6. At 3 weeks postoperatively cystography is performed. If the VVF is healed and the patient voids to completion after the removal of the urethral catheter, the suprapubic tube, if *in situ*, is removed. If there is still leakage on cystography, catheter drainage is recommended for an additional period. Persistent leakage at 6 week requires repeat operative repair.
7. The patient is counselled to avoid vaginal intercourse for 3 months after the operation.

**Table 1.** Considerations for successful repair of VVF.

Preoperative considerations
1. Timing of repair
2. Approach: vaginal vs abdominal
3. Health of tissues
a. Oestrogenisation
b. RT
4. Condition of the patient:
a. Anaemia
b. Hypoalbuminaemia
5. Concomitant surgical procedures
a. SUI surgery
b. Prolapse surgery
c. Augmentation cystoplasty
d. Ureteric surgery
**Intraoperative considerations**
a. Good exposure of fistulous site
b. Wide mobilisation of tissues
c. Good closure:
– Tension-free
– Watertight
– Multilayer repair
– Non-overlapping suture lines
d. Tissue interposition
e. Bladder drainage; sometimes faecal diversion may be required
**Postoperative considerations**
a. Avoidance of infection
b. Close monitoring to ensure sound bladder drainage
c. Adequate oestrogenisation
d. Prevention of bladder spasms (anticholinergics)

## Conclusions

The transvaginal approach is a simple procedure, less invasive than the abdominal approach, and is associated with less morbidity and blood loss. For successful repair of the VVF, the surgeon must consider all factors related to the case: preoperative, intraoperative, and postoperative considerations. Recognition of possible injury to the bladder during a gynaecological operation should be thoroughly investigated and managed immediately with the proper technique. There are no randomised studies available that directly compare the outcomes of transabdominal vs transvaginal approaches. There is no strong evidence to support the necessity for routine use of grafts in VVF repair. The use of grafting in obstetric VVF has significantly declined.
